# Diagnosing Lassa virus infection by tracking the antiviral response

**DOI:** 10.1186/1471-2105-13-S18-A13

**Published:** 2012-12-14

**Authors:** Ignacio S Caballero, Gracia Bonilla, Judy Y Yen, John H Connor

**Affiliations:** 1 Department of Bioinformatics, Boston University, MA, USA; 2 Department of Microbiology, Boston University, MA, USA

## Background

Lassa fever is an acute viral hemorrhagic fever caused by the Lassa virus. It infects 300,000 to 500,000 West Africans every year, with a mortality rate among hospitalized patients of 15%. It is difficult to diagnose because its early symptoms (fever, sore throat, general malaise) often go unnoticed, or are confused with those of the common flu, malaria, or other febrile diseases. For this reason, there is considerable interest in developing tests that can detect the presence of the virus at the earliest stages of infection, when treatment is most effective. Methods such as the enzyme-linked immunosorbent assay (ELISA) or the real-time polymerase chain reaction (RT-PCR) are commonly used to detect virus particles in the blood, but in the case of Lassa, when the infection becomes detectable, it is already too late to administer treatment.

In this study, we took an indirect approach and analyzed the transcriptional response of the circulating immune system, instead of directly measuring the amount of virus in the blood, to see if we could detect earlier signs of infection. We extracted samples of peripheral blood mononuclear cells from infected non-human primates at sequential timepoints for a period of 12 days. The RNA in each sample was assayed in a microarray to quantify the transcriptional pattern followed by each gene throughout the infection. We used these patterns to identify genes with the strong transcriptional changes 3 days after infection, since these have the greatest chance of being biologically significant, and could be used as early biomarkers of infection.

## Results

**Figure 1:**
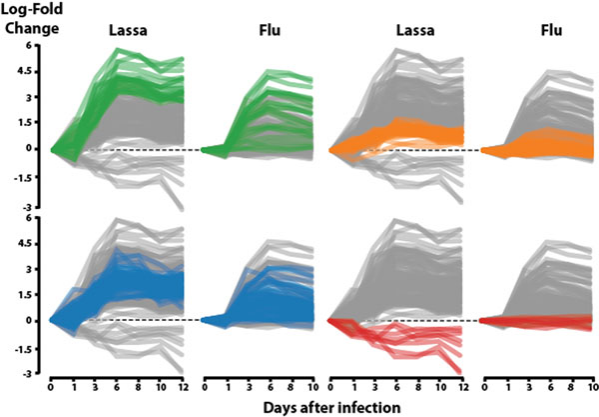
Expression patterns of 150 significant genes throughout Lassa and flu infection, using their pre-infection samples as baseline. Genes are colored based on the Lassa cluster they belong to. The majority of genes follow a similar pattern in both types of infection, with the exception of those in the green and red clusters.

Out of the ~150 genes that we identified as differentially expressed, many haven't been described to play any significant role in immunity, but others involved in the JAK-STAT and the interferon pathways are well known regulators of the antiviral response. To further limit our list of candidate biomarkers, we compared it with another set of genes obtained from a similar experiment conducted on patients that were infected with the common flu [1]. This allowed us to remove genes that showed similar patterns of expression in both diseases, indicating that they have little discriminative power, and keep those that were characteristic of Lassa virus infection (Figure 1 ).

## Conclusions

We are in the process of acquiring additional samples to validate the specificity and sensitivity of our biomarker, with the medium-term goal of applying it in a clinical setting. We believe we have identified a gene signature that could potentially diagnose Lassa fever infection in patients before they show specific clinical symptoms, an essential requirement in treatment and prevention.
